# Strengthening the role of community pharmacy in HPV vaccination roll-out in Serbia at national and local levels: A pharmacy-based education approach

**DOI:** 10.1371/journal.pone.0322584

**Published:** 2025-04-29

**Authors:** Ivana Rapajić-Moran, Brankica Filipić, Dragana Rajković, Milan Rakić, Dragana Stojiljković, Bojana Letić, Jasna Urošević, Nataša Bogavac-Stanojević

**Affiliations:** 1 Department of Social Pharmacy and Pharmaceutical Legislation, Doctoral Studies, University of Belgrade-Faculty of Pharmacy, Belgrade, Serbia; 2 Department of Microbiology and Immunology, University of Belgrade-Faculty of Pharmacy, Belgrade, Serbia; 3 The Pharmaceutical Chamber of Serbia, Belgrade, Serbia; 4 Department of Medical Biochemistry, University of Belgrade-Faculty of Pharmacy, Belgrade, Serbia; Ogun State College, College of Nursing, NIGERIA

## Abstract

Cervical cancer is a significant public health concern in Serbia, with high morbidity and mortality rates (27 and 14.2 per 100,000 women, respectively in 2020). The primary cause of cervical cancer is human papillomavirus (HPV) infection, and HPV vaccination has proven to be an effective prevention strategy. This publication discusses the implementation of the first pharmacy-based education (PBE) program realized through the project of Pharmaceutical Chamber of Serbia – “Ask me about HPV” which aimed at raising awareness of the general population about the importance of HPV vaccination. The program aimed to strengthen the role of community pharmacists in the HPV vaccination roll-out. A total of 250 pharmacists were selected and trained to provide information on HPV infection and promote vaccination uptake. The program aimed to counsel and to educate young adults and parents/guardians. A total of 24,327 subjects were educated during the program’s implementation from February to May 2023, young adults (N=11,313) and parents/guardians (N=13,014). Data showed that only 4.9% of young adults and 6.4% of children (of the parents/guardians surveyed) in Serbia are vaccinated against HPV. The questionaries of 1,387 subjects already vaccinated were excluded from the further analysis and final analysis was performed based on the counseling of 22,941 participants. A significantly higher percentage of parents/guardians stated that they would have their children vaccinated against HPV after counseling at the pharmacy than young adults (42.3% and 34.1% respectively). On the other hand, of those respondents who stated they would get vaccinated after consulting at the pharmacy, a significantly higher number of young adults (51%), compared to parents/guardians (48%), would receive the vaccine specifically at the pharmacy. The results of the project indicate that education of the general population on the benefits of HPV vaccination is highly needed. The expected outcomes of this educational program are increased awareness of the burden of illness, improved consumer education, the potential for higher vaccination rates, and consequently a reduction in cervical cancer incidence and mortality in Serbia.

## Introduction

Cervical cancer is a significant public health concern in Serbia, with high morbidity and mortality rates (27 and 14.2 per 100,000 women, respectively in 2020). In Serbia, cervical cancer is the third most common cancer in women, after breast and colorectal cancer and the country occupies third place in Europe regarding the mortality rates due to cervical cancer, after Romania and Montenegro [1,2]. The Southeast European region is at the top of the charts in Europe in the incidence and mortality of cervical cancer, and this important public health problem is a major health issue in Serbia, with a high number of cases and deaths recorded each year.

The primary cause of cervical cancer is human papillomavirus (HPV) infection, and HPV vaccination has proven to be an effective prevention strategy [3,4]. In addition to cervical cancer, HPV causes other anogenital cancers (anal, vulvar, vaginal, penile) and head and neck cancers [5,6]. To lower the incidence of HPV-related cancers, the World Health Organization (WHO) recommends introduction of the routine vaccination against HPV through the National Immunization Program (NIP) [7].

Routine HPV vaccinations are available globally, in accordance with local regulations via the National Immunization Programs (NIPs), Reimbursement Framework Programs (RFPs), or through private clinics of eligible specialists who can prescribe them. These programs provide vaccines at no cost to pediatric and adolescent populations, with the age range of eligible individuals varying by country; in some places, like Serbia, this may include children aged 9–19 years [8,9]. This publication discusses the implementation of the first pharmacy-based education program (PBE) realized through the project of the Pharmaceutical Chamber of Serbia – “Ask me about HPV” which aimed at raising awareness of the general population and consumers about the importance of HPV vaccination in Serbia. The accredited education program for the selected pharmacists commenced in January 2023, seven months after the NIP commencement, and this pilot PBE program was rolled out from February 1 until May 31, 2023. The “Ask me about HPV” project implemented by the Pharmaceutical Chamber of Serbia through member pharmacies across the country, aimed to strengthen the role of community pharmacies in the HPV vaccination roll-out. The project focused on regions with high cervical cancer incidence rates, where disparities in healthcare infrastructure, access and information exist. The aim of the study was twofold: first, to examine the awareness of HPV infections and HPV vaccination among two distinct groups, namely young adults and parents/guardians of unvaccinated children; and second, to analyze the differences in awareness according to gender.

As far as we know, this is the unique and largest PBE-based project supporting HPV vaccination in Serbia, with comprehensive analyses of the attitudes of young adults and parents/guardians regarding HPV vaccination acceptance.

The PBE program implemented by trained pharmacists is essential for pharmacy-based vaccination (PBV) programs [10,11]. Research indicates that PBV programs can enhance vaccination rates, particularly in underserved populations and areas with limited healthcare resources [12,13]. Implementing PBV programs during heightened public health concerns, such as the COVID-19 crisis, can maximize the uptake of critical prevention strategies [14,15]. Moreover, effective PBE and PBV programs may alleviate the burden on primary healthcare, allowing physicians to focus more on patient education and improving health outcomes [16,17].

## Methods

### Selection of community pharmacists

To be eligible for participation in the “Ask me about HPV” project, applicants had to be licensed pharmacists registered with the Pharmaceutical Chamber of Serbia and working in community pharmacies. In 2023 the “Ask me about HPV” project consisted of selecting, training and educating 250 pharmacists in the Republic of Serbia to promote HPV vaccination awareness, with a special focus on 5 regions where the incidence rate of cervical cancer was higher than 24 per 100,000 inhabitants: North Banat Region, West Backa Region, Zajecar Region, Bor Region and Nisava Region. The 250 pharmacists have been selected based on the potential of the project realization. Two hundred and fifty pharmacists have been selected prospectively as well as 5 regional coordinators. Each regional coordinator was responsible for approximately 50 pharmacists in their respective regions. The initial idea of the project was to primarily implement it in the specified 5 regions, which is why we defined 250 pharmacists (50 pharmacists per region). To ensure all pharmacists had an equal opportunity to participate, a public call for participation in the project was opened. Since 250 pharmacists did not apply from the specified regions (14 applied from Nort Banat Region, 33 form West Backa Region, 21 form Zajecar Region, 18 from Bor Region, 51 from Nisava Region) and there was significant interest from other places/cities, the project included pharmacists from other regions as well. The distribution of pharmacists from other regions was 35 from South Backa Region (including Novi Sad), 16 from Belgrade District (capital of Serbia), 12 from North Backa and Central Banat (12), Sumadija District (7), South Banat (6), Pomoravlje District (5), Srem District (4), Pcinja District (4), Macva District (3) and other regions (9). Of the 250 pharmacists included in the study, 234 were from urban regions and 16 were from rural regions. Although the number of pharmacists from rural areas is small at first sight, it should be emphasized that people from rural areas are usually oriented towards urban regions for jobs, medical treatments, etc., meaning that they could also be part of the counselling in urban areas. The incidence and mortality rates of cervical cancer per 100,000 population by different districts in the Republic of Serbia is among the highest in Europe [18].

Eligible pharmacists were selected through interview process which consisted of two calls. The first call for pharmacists was from October 2, to November 1, 2022. The call was announced in the publication by the Pharmaceutical Chamber of Serbia „Apotekarska praksa“ No. 133, and sent by email to all members of the Pharmaceutical Chamber of Serbia. The second call was from November 10, to November 20, 2022, and sent directly by email to all members of the Pharmaceutical Chamber of Serbia.

During the selection of applicants, preference was given to pharmacists who work in the regions with the highest incidence of cervical cancer, as well as pharmacists who work in pharmacies within the health centers, or near the health centers, for easier communication and direct referral for vaccination. This preference, however, did not exclude the participation of pharmacists from other regions in the country. The final list of participants included 250 pharmacists, of which 120 (48%) were from the regions with the highest rate of cervical cancer (North Banat Region - 13, West Backa Region - 20, Zajecar Region - 29, Bor Region - 5, Nisava Region - 53).

### Education of pharmacists included in the project

Selected pharmacists received comprehensive education on January 25, 2023, covering various aspects related to HPV, including detailed information on HPV itself, modes of viral transmission, potential complications associated with HPV infections, advantages of HPV vaccination, efficacy and safety of the vaccine, as well as potential adverse effects. The training program also included relevant topics such as improving communication skills for adequate patient counseling and helping patients deal with misinformation and overcome vaccine hesitancy. All education and training programs were conducted by professors from relevant academic sectors, ensuring that the information provided was based on the most up-to-date scientific data available. During the online education session, the questionnaires for young adults and parents/guardians were introduced to the participants. This training was provided by five leading pharmacists who coordinated subgroups of pharmacists during project implementation.

Participants were provided with recorded presentations given by university professors, along with written materials to support the education provided. The mandatory final step of the training was a test consisting of 60 questions, which every trained pharmacist was required to pass.

After completing the training, pharmacists were awarded a badge that designated them as “Vaccination Advisors for HPV”. This badge served as a recognition of their acquired knowledge and skills, indicating their ability to effectively provide information about HPV and HPV vaccination to a diverse range of target groups, such as adolescents, students, and young adults under 26 years of age, as well as parents, or guardians.

### Implementation of the project

A total of 250 recruited pharmacists were organized into five groups, assigned a dedicated contact pharmacist available to address any questions or uncertainties related to counseling. The five designated as contact pharmacists also engaged with academic educators to clarify any doubts or correct misinformation.

All pharmacists who participated in the project followed standardized questionnaires designed separately for young adults (19–26 years, S1 Material) and for parents/guardians of children (9–18 years, S2 Material), to assess patients’ needs and provide reliable scientific information about HPV and vaccination. The age categories were chosen based on the fact that vaccination in Serbia is free for the age group 9–19 years, and there are plans to make free vaccination available to young adults who are, in any case, the target group for the HPV vaccine. Children themselves were not involved in answering the questionnaires - instead, their parents/guardians did it for them. The questionnaires were designed to assess awareness and attitudes about HPV, HPV-related disease and HPV vaccine, while also providing evidence-based information about HPV vaccination. The questionnaires were designed as checklists (S1 and S2 Materials) to assist pharmacists in providing counseling and ensuring that all essential information about HPV vaccination is communicated to young adults and their parents/guardians. The questionnaires were completed by pharmacists, serving primarily as a guide to help them determine which questions to ask the patient, assess the patient’s prior knowledge of HPV and HPV vaccination, and identify areas where additional information about HPV vaccination was needed. The use of uniform questionnaires, followed by pharmacists during the project implementation, enabled standardization of the counseling in different regions of Serbia. Pharmacists also contributed to the development of individuals’ HPV Vaccination Action Plans.

By providing accurate and evidence-based information, pharmacists helped individuals make informed decisions about HPV vaccination and address any misconceptions or doubts they may have had. The project’s expected outcomes included increased vaccination rates, improved access to healthcare services, and the development of a pool of HPV-trained pharmacists who can continue to promote vaccination and provide education within their communities. The project’s impact is anticipated to continue in contributing to the reduction of HPV-related cancers incidence and mortality in Serbia.

### Subjects included in the study

Data was collected using uniform questionaries by 250 pharmacists in 211 Pharmacies. Counseling, monitored through uniform questionnaires, was conducted in pharmacies marked with a poster that read “Ask me about HPV”. Additionally, trained pharmacists wore badges with the same message -“Ask me about HPV”. Counseling was initiated either by the patient/service user or the pharmacist. So, the trained pharmacists could initiate a conversation with the patient about HPV vaccination, or interested patients could start the conversation with pharmacists who wore a badge that read “Ask me about HPV”. Counseling was conducted from February 1 until May 31^st^, 2023, and all participants (young adults and parents/guardians) who were counseled agreed to participate in the survey by signing a written informed consent which was waived by the Ethics Committee of the Pharmaceutical Chamber of Serbia (Ethical approval No. 316/6-4-6). The written informed consent was personally signed by participants over the age of 18, while for those under the age of 18, parents/guardians were involved in the counseling and the informed consent was signed by parent/guardian.

Educated pharmacists provided counseling to young adults and students (N=11,313) and parents/guardians (N=13,014) in different regions of the country – a total of 24,327 subjects were included in the counseling during the program′s implementation. The questionaries of 1,387 subjects were excluded from the further analysis as these individuals were already vaccinated against HPV. Final analysis was performed based on the counseling of 22,941 participants.

All collected data of this retrospective study were accessed for the research on 20/12/2023 and the authors did not have access to information that could identify individual participants.

### Statistical analysis

Data obtained using uniformed questionaries were analyzed using appropriate statistical methods. Different age of selected populations was presented as mean ± standard deviation and were compared by Student t-test for two independent populations. Categorical variables were shown as relative and absolute frequencies and analyzed using the Chi-square test for association. A two-sided p-value of p ≤0.05 was considered significant. Analyses were conducted by the statistical program SPSS 27.0 (SPSS Inc. Chicago, USA).

## Results

Within the “Ask me about HPV” project, a total of 24,327 people took part in the study. Forty-six percent of the participants were adolescents aged 18 and 26 years (young adults), (11,313/24,327) and 54% of the participants (13,014/24,327) were parents or guardians of children under the age of 18 (parents/guardians). Among the counselled population, only 4.9% (554/11,313) of young adults and 6.4% (832/13,014) of children of counselled parents/guardians were vaccinated against HPV, and most of them were female, with young women among the group of young adults being more frequently vaccinated 81.8% (453/556), than girls among the group of counselled parents/guardians 72.5% (601/829), p<0.001.

The questionnaires of participants who responded in the survey that they were vaccinated (N=1,387) were not further included in the analysis, while the questionnaires of unvaccinated young adults (N=10,757, Table 1) and the questionnaires filled out by parents/guardians (N=12,184, Table 1) of unvaccinated children were included for further analysis.

**Table 1:** Demographic characteristics and awareness of young adults and parents/guardians about HPV and HPV vaccine.

	Total N=22,941	Young adults N=10,757	Parents/guardians N=12,184	P
**Females, % (N)**	60.1% (13,794)	65% (6,990)	55.8% (6,804)^#^	<0.001
**Males, % (N)**	39.9% (9,147)	35% (3,767)	44.2% (5,380)	
**Age, years**	16.1±2.8	20±3.0	12.6±2.7^#^	<0.001^*^
**Do you know what HPV is?**				
** Yes**	33.7%	32.5%	34.7%	<0.001
** Partially**	44.9%	44.1%	45.7%	
** No**	21.3%	23.4%	19.5%	
**Do you know what problems HPV causes?**				
** Yes**	25.4%	25.1%	25.7%	<0.001
** Partially**	50.7%	48.6%	52.5%	
** No**	23.8%	26.3%	21.7%	
**Do you know about the HPV vaccine?**				
** Yes**	37.6%	38.8%	36.7%	<0.001
** Partially**	31.9%	28.6%	34.9%	
** No**	30.3%	32.6%	28.3%	
**Are you concerned about the HPV vaccination?**				
** Yes**	20.8%	15.5%	25.6%	<0.001
** Partially**	43.4%	40.0%	46.8%	
** No**	35.4%	44.5%	27.6%	

*Difference between young adults and parents compared by Student t-test.

#Data for children are presented.

The average age of the people who received counseling was 20±3.0 years for young adults (age group 19–26) and 12.6±2.66 years for children (age group 9–18) whose parents/guardians were interviewed. Significantly more young women, 65% (6,990/10,757) were counselled than 55.8% (6,804/12,184) parents/guardians of girls (p<0.001).

Most of the study participants were partially informed about the HPV and the problems associated with HPV infection (44.1% of young adults and 45.7% of parents/guardians, Table 1). However, the highest percentage of respondents indicated that they were aware of the HPV vaccine availability (38.8% of young adults and 36.7% of parents/guardians, Table 1). A lower percentage of parents were unaware of the HPV and vaccination than young adults (28.3% and 32.6%, respectively), and they were more concerned about HPV vaccination compared to young adults (25.6% and 15.5%, respectively, Table 1).

A significantly larger percentage of parents/guardians indicated that they would have their children vaccinated against HPV following counseling at the pharmacy, compared to young adults, with rates of 42.3% (5,154/12,184) and 34.1% (3,668/10,757), respectively (Table 2). Only 4% (487/12,184) of parents/guardians would refuse to have their children vaccinated, compared to 7.3% (785/10,757) of young adults. Overall, 84.5% of participants in both groups stated that they trust healthcare professionals (doctors and pharmacists) the most. Other sources of information, such as: social network, internet, media and people close to you, are represented by a lower percentage (Table 2). However, significantly more parents/guardians believe in healthcare professionals than young adults, 89.1% (10,855/12,184) vs 81.2% (8,831/10,7570) respectively (Table 2).

**Table 2:** Opinion about HPV vaccination after pharmacy counseling service and source of information.

	Total N=22,941	Young adults N=10,757	Parents/guardians N=12,184	P^*^
**Would you or your children receive the HPV vaccine after pharmacy counseling service?**				
** Yes**	38.2%	34.1%	42.3%	<0.001
** No**	5.5%	7.3%	4.0%	
** Not now**	34.2%	31.2%	37.3%	
** I am not sure**	21.4%	27.4%	16.4%	
**Which sources of information do you trust the most?**				
** Social network**	1.8%	3.0%	0.9%	<0.001
** Internet**	3.5%	4.9%	2.4%	
** Media**	1.2%	1.1%	1.3%	
** People close to you**	8.5%	9.9%	7.3%	
** Healthcare workers** ^*^	84.5%	81.2%	89.1%	

*Difference between young adults and parents/guardians by chi-square test.

Responses regarding the HPV and the HPV vaccination were compared between genders in each group (young adults and parents/guardians) and the results are shown in Table 3. A partition of respondents by gender showed that a significantly higher percentage of men and parents/guardians of boys were uninformed about the HPV and associated problems and about the HPV vaccination than young women and parents/guardians of girls (Table 3).

**Table 3:** Awareness of the HPV and the HPV vaccination among male and female young adults and parents/guardians of boys and girls.

	Young adults N=10,757		Parents/guardians N=12,184	
	Men (N=3,767)	Women (N=6,990)	Boys (N=3,767)	Girls (N=7,531)
**Do you know what HPV is?**		*		*
** Yes**	21.7%	38.4%	33.7%	35.6%
** Partially**	41.6%	45.4%	44.5%	46.8%
** No**	36.7%	16.2%	21.9%	17.7%
**Do you know what problems HPV causes?**		*		*
** Yes**	17.0%	29.5%	24.6%	26.6%
** Partially**	43.9%	51.2%	51.6%	53.5%
** No**	39.2%	19.3%	23.9%	20.0%
**Do you know about the HPV vaccine?**		*		*
** Yes**	27.0%	45.1%	34.6%	38.4%
** Partially**	27.3%	29.2%	34.6%	35.2%
** No**	45.6%	25.6%	30.8%	26.4%
**Are you concerned about the HPV vaccination?**		*		*
** Yes**	15.5%	15.5%	24.8%	26.2%
** Partially**	37.6%	41.3%	46.6%	46.9%
**No**	47.0%	43.2%	28.5%	26.9%

*Significant difference between genders (p<0.001).

The highest percentage of respondents who would refuse to be vaccinated in the future were young men, and they were significantly more prevalent than young women (11.1% (418/3,767) vs 5.3% (370/6,990), p<0.001 (Table 4). The difference was also significant in the group of parents/guardians, but only 4.4% (166/3,767) and 3.6% (271/7,531) of parents/guardians of boys and girls, respectively, would refuse to have their children vaccinated (Table 4). Healthcare professionals were identified as the most trusted source of information among parents/guardians in the study, and the gender of the children did not have an impact on this perception of trust. However, a significantly higher percentage of young women trusted healthcare professionals more than young men (Table 4).

**Table 4:** Gender differences regarding vaccination after pharmacy counseling and source of information in groups of young adults and parents/guardians.

	Young adults N=10,757		Parents/guardians N=12,184	
	Men (N=3,767)	Women (N=6,990)	Boys (N=3,767)	Girls (N=7,531)
**Would you receive the HPV vaccine after pharmacy counseling service?**		*		*
** Yes**	26.9%	37.9%	39.2%	44.8%
** No**	11.1%	5.3%	4.4%	3.6%
** Not now**	30.0%	31.9%	38.5%	36.3%
** I am not sure**	32.1%	24.9%	17.9%	15.2%
**Which sources of information do you trust the most?**		*		
** Social network**	4.3%	2.2%	0.8%	0.9%
** Internet**	7.3%	3.6%	2.9%	1.9%
** Media**	1.5%	8%	1.4%	1.3%
** People close to you**	12.6%	8.4%	8.3%	6.6%
** Healthcare workers**	74.2%	84.9%	86.6%	89.3%

*Significant difference between genders (p<0.001).

**Fig 1:**
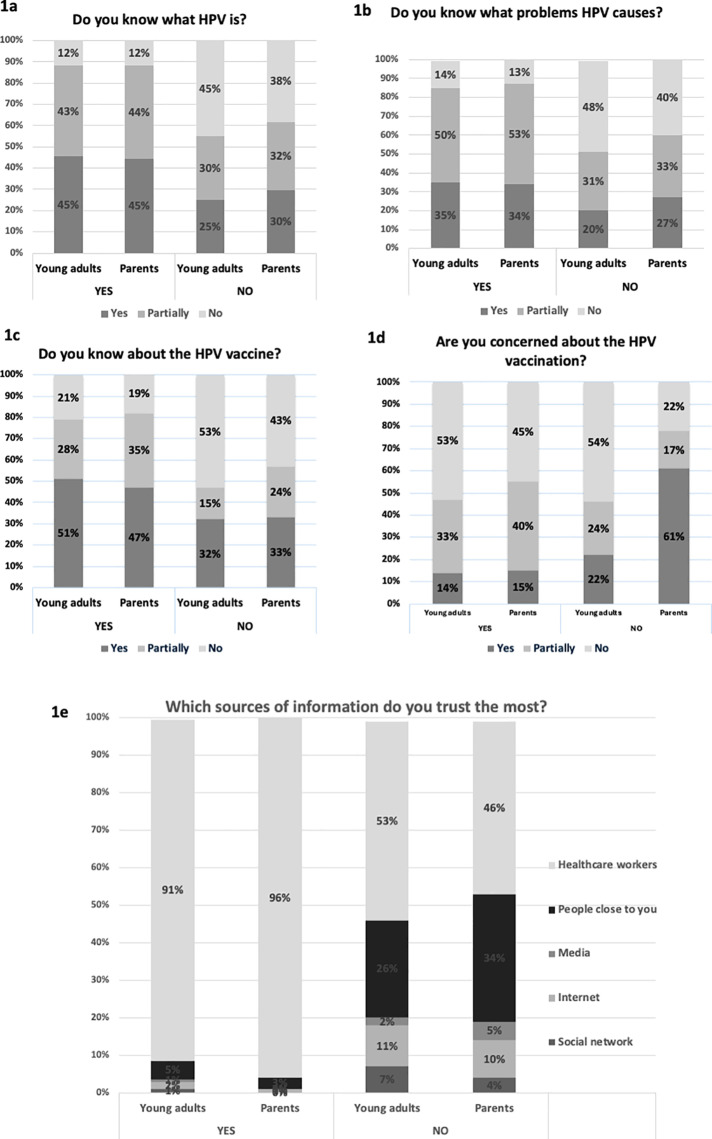
The relationship between prior knowledge/attitudes about HPV and HPV vaccine and the decision (Yes/No) about vaccination after pharmacy counseling service. a - impact of the prior knowledge about HPV on the decision about vaccination (Yes/No); b - prior knowledge about problems that HPV causes; c - prior awareness about HPV vaccine; d - prior concerns about the HPV vaccination; e - relation between sources of information and decision about HPV vaccination.

The relationship between prior knowledge about HPV and the decision about future vaccination was analyzed and results are shown in S1 Table and Fig 1.

Decisions about vaccination after counseling in the pharmacy depended on prior knowledge about the HPV vaccine and associated problems in a group of young adults. The lowest percentage of young adults who would get vaccinated was in the group of those who were unaware of HPV and the problems associated with it (12% and 14%, respectively) (Fig 1a and Fig 1b). A similar pattern was seen in the group of parents/guardians but only in terms of prior knowledge about HPV (Fig 1a). Uninformed parents/guardians about HPV were less likely to have their children vaccinated than those who were better informed (p=0.004) (Fig 1a). Knowledge about HPV vaccine was also related to the decision in favor of vaccination in both groups (Fig 1c). When higher knowledge was available, more respondents decided in favor of vaccination or wanted to have their children vaccinated (Fig 1c). However, if concerns about HPV vaccination increase, fewer young adults will be vaccinated, and fewer parents/guardians will vaccinate their children (Fig 1d). Respondents who would refuse to be vaccinated or have their children vaccinated had less confidence in healthcare professionals than the rest of the respondents (Fig 1e). In this group, 26% of young adults and 34% of parents/guardians have the most trust in people close to them, significantly more than the other respondents (Fig 1e).

The opinions of young adults and parents/guardians about HPV vaccination in pharmacies were also analyzed. This information is important because vaccination at the pharmacy is still not approved in Serbia. Only those participants who stated that they or their children would receive the HPV vaccine after counseling in pharmacy were analyzed (34.1% (3,668) of young adults and 42.3% (5,153) of parents/guardians, Table 2). Around half of the respondents would be vaccinated against HPV at the pharmacy. Significantly more young adults were positive about being vaccinated at the pharmacy and not in another healthcare institution 51% (1,871/3,668) than parents/guardians 48% (2,495/5,153), p<0.001.

## Discussion

Since June 2022, the nonavalent HPV vaccine Gardasil 9™ has been made available free of charge to girls and boys aged 9–19 years through the Republic Fund of Health Insurance in Serbia, recognizing that the HPV vaccination is the most effective measure for preventing HPV-related cancers in both women and men. The project presented in this study, “Ask me about HPV” of the Pharmaceutical Chamber of Serbia is the first implemented PBE project related to HPV vaccination to strengthen the role of community pharmacists in the HPV vaccination roll-out and to improve vaccination rates, particularly in regions of Serbia with high incidence of cervical cancer. Previous research has found that a variety of interventional strategies promoting HPV vaccination increase its uptake [19,20].

The “Ask me about HPV” was the largest project of the Pharmaceutical Chamber of Serbia so far, with 250 trained pharmacists and counseling of 24,327 individuals in Serbia on the benefits of the HPV vaccination – 11,313 adolescents and students aged 18–26 (young adults) and 13,014 parents, or guardians of children aged 9–17. According to the data obtained during the project, only 4.9% of young adults and 6.4% of children (of the parents/guardians surveyed) in Serbia are vaccinated against HPV-related illnesses and malignancies. This low vaccination coverage rate (VCR), unfortunately, corresponds to the real available data, where only 734 girls and boys 9–14 years old have received two doses, while 618 girls and boys aged 15–19 have received three doses of the HPV vaccine in Serbia by the end of 2022 [21].

These VCRs in Serbia are amongst the lowest VCRs in the region and Europe. As Serbia ranks third in Europe with a cervical cancer mortality rate of 14.2 per 100,000 women [2], comprehensive vaccine promotion strategies are urgently needed.

A similar study conducted in Croatia, former Yugoslavian country, showed that 18.3% of participants from 1,197 individuals included in the study were HPV vaccinated, while 65.6% of those vaccinated were women [22]. Although the results in Croatia are somewhat better than those in Serbia, there is still the need to increase HPV vaccination uptake in Croatia through raising awareness about HPV vaccine effectiveness [22]. On the other hand, in Slovenia, HPV vaccination was implemented in 2007 and among eligible girls gradually increased and reached 59.3% in the 2018/19 school year, HPV vaccination program was expanded to boys 11–12 years old and coverage for two doses of HPV vaccine reached 14.73% in the 2021/22 school year among eligible boys [23].

All three studies from former Yugoslavian countries (Serbia, Croatia, and Slovenia) indicate three possible conclusions: a) HPV vaccination is significantly higher among females (in this study 81.8% (453/556) females among young adults and 72.5% (601/829) girls/children of the parents/guardians surveyed and women’s counseling outnumbered men’s counseling); b) the most trusted source of vaccination information are healthcare providers (particularly for parents/guardians in this study-89.1% (89.3% and 86.6% for parents/guardians of girls and boys respectively) comparing to young adults-81.2% (84.9% and 74.2% for women and men respectively)); c) it is necessary to disseminate information about the benefits and safety of HPV vaccination to the widest possible range of health professionals and to ensure that their knowledge is continuously updated and widely disseminated to the general public using well-planned educational campaigns [22–24].

It can be concluded that the importance of the HPV vaccination in males is underestimated especially knowing that HPV is highly prevalent globally with 1 in 3 men and 1 in 10 women are infected in their lifetime [25]. HPV infection is commonly associated with cervical cancer, which is why women are usually more informed about the virus and vaccination. According to our study, significant gender differences were observed in knowledge about HPV (p<0.001). Specifically, 38.4% of women and 21.7% of men, as well as 35.6% of girls and 33.7% of boys (of the parents/guardians surveyed), demonstrated awareness of HPV. Additionally, 29.5% of women and 17% of men, along with 26.6% of girls and 24.6% of boys (of the parents/guardians surveyed), were familiar with the health issues caused by HPV. These data revealed that a significantly less percentage of men and parents/guardians of boys were informed about the HPV, associated problems and HPV vaccination than young women and parents/guardians of girls. These results are not in line with the survey study conducted in European countries with a longer history of HPV vaccination. A literature review study on the acceptability of male HPV vaccination was carried out in the UK, Germany and Italy where approximately ¾ of parents were in favor of HPV vaccination of their sons [26].

The “Ask me about HPV” project has demonstrated the crucial role that pharmacists play in educating and counseling the general population. The findings revealed that many study participants had only partial knowledge about HPV, with 44.1% of young adults and 45.7% of parents/guardians reporting limited awareness, and 48.6% of young adults and 52.5% of parents/guardians being partially informed about the issues linked to HPV infection. Highlighting the importance of pharmacists in counseling, 42.3% of parents/guardians stated that they would have their children vaccinated against HPV after receiving counseling at the pharmacy. However, the percentage of parents/guardians stated that they would have their children vaccinated against HPV after counseling at the pharmacy (42.3%) was significantly higher than among counselled young adults (34.1%). Similarly, in another online, 4-group, randomized controlled trial, with 260 or more participants per group, was found that parents of children were more likely to accept HPV vaccination than were young adults for themselves (68.2% and 52.9%) [27]. The possible reason for these differences could be explained by the fact that the HPV vaccination program is mainly focused on children whose parents gave consent to vaccination, so the success of the HPV vaccination program depends on the parents’ decision. Having this in mind, greater efforts are invested in the education of parents compared to young adults and additionally, pediatricians’ knowledge and attitudes toward vaccination can significantly influence the decision regarding HPV vaccination in parents [28].

Altogether, these results present encouraging findings for the improvement of VCRs in the future in Serbia and implementation of the “Ask me about HPV” project represents a small, but significant step forward towards Pharmacy Based Vaccination (PBV), which has proven to become a successful public health measure in many countries around the world [29–32].

## Conclusions

The pharmacy-based education program ‘Ask me about HPV’ has demonstrated effectiveness in enhancing awareness of HPV- related burden of illness, and the importance of HPV vaccination among two distinct groups: young adults and parents/guardians. This initiative has the potential to contribute positively to public health outcomes. Additionally, the project identified significant gender differences in awareness; women and parents/guardians of girls displayed greater knowledge compared to men and parents/guardians of boys.

The “Ask me about HPV” project in Serbia demonstrated the significance of involving community pharmacists in the implementation of HPV vaccination programs. By providing pharmacists with the necessary knowledge, skills, and resources, this project successfully enhanced the role of community pharmacies in promoting HPV vaccination, aiming to reduce vaccine hesitancy, clarify misconceptions and provide accurate information to the involved participants. Counseling laid the groundwork for future strategies aimed at reducing the incidence and mortality of cervical cancer in the country.

## Limitations

The potential limitation of this study is that questionnaires were filled out by pharmacists which may impact the study’s results, influencing factors such as response bias, accuracy, and data consistency. On the other hand, the questionnaires primarily served as a guide to help pharmacists determine which questions to ask the patient, assess the patient’s prior knowledge of HPV and HPV vaccination, and identify areas where additional information on HPV vaccination needed to be provided during counseling. In addition, to overcome this potential limitation, participants (pharmacists) were divided into five groups, each with their own contact pharmacist who could address any doubts and questions regarding counseling. The five pharmacists who served as contact points also had communicated with all educators from academia to address any doubts or misinformation.

## Supporting information

S1 Material Questionnaire for young adults. (PDF)

S2 Material Questionnaire for parents/guardians. (PDF)

S1 Table The relationship between prior knowledge about HPV and the decision about vaccination. (PDF)
