# Supplementary material for: Strengthening the role of community pharmacy in HPV vaccination roll-out in Serbia at national and local levels: A pharmacy-based education approach
Source: PLoS One. 2025 Apr 29;20(4):e0322584. doi: 10.1371/journal.pone.0322584 (PMC12040191; doi:10.1371/journal.pone.0322584)
Supplement: S1 Material — (PDF) [file pone.0322584.s001.pdf]

Questionnaire for young adults

1. **Have you already received the HPV vaccine?**

Yes. No

If "NO", provide counseling service. If the answer is "YES", record that answer and no further service is required.

2. **Do you know what Human papilloma virus (HPV) is?**

Yes Partially NO

Indicate what advice/information you have given to the service user:

a) What is HPV;

Yes No

b) How HPV is transmitted;

Yes. No

c) How HPV infection can be prevent;

Yes. No

3. **Do you know what problems HPV causes?**

Yes Partially No

Indicate what advice/information you have given to the service user:

-a) what are the possible consequences of HPV infection;

Yes. No

b) do all women/men with HPV infection get cancer;

Yes, No

c) how common is cancer caused by HPV infection;

Yes No

4. **Do you know about the HPV vaccine?**

Yes Partially No

Indicate what advice/information you have given to the service user:

a) why HPV vaccination is important;

YES. NO

b) Can people of both sexes be vaccinated;

YES. NO

c) How many doses of HPV vaccine are needed depending on gender;

YES. NO

d) Is it important that the person receives all the recommended doses of the vaccine;

YES. NO

e) at what age three doses of the HPV vaccine are received;

YES. NO

f) how long protection lasts after complete HPV vaccination;

YES. NO

g) will the HPV vaccination prevent the vaccinated person from getting cancer;

YES. NO

h) whether a person can get an HPV infection after HPV vaccination;

YES. NO

i) where the HPV vaccine can be received;

YES. NO

j) if a person already has an HPV infection, can the vaccine lead to a cure (to eliminate HPV from the body);

YES NOT APPLICABLE NO

k) whether regular gynecological/urological examinations are required after vaccination;

YES. NO

-l) the possibility of HPV vaccination of persons with a disorder of the immune system/immunosuppressive disease;

YES NOT APPLICABLE NO

5. Are you concerned about the HPV vaccination?

YES PARTIALLY NO

Indicate what advice/information you have given to the service user:

a) The HPV vaccine is safe and effective;

YES. NO

b) HPV vaccination does not affect fertility;

YES. NO

c) HPV vaccine safety during pregnancy and breastfeeding;

YES NOT APPLICABLE NO

d) effectiveness of the HPV vaccine in sexually inactive/active persons;

YES. NO

e) the vaccine is equally effective in men and women;

YES. NO

f) contraindications for vaccination;

YES. NO

g) the reason for the possible occurrence of fainting after HPV vaccination;

YES. NO

h) the safety of vaccination if the person previously had an adverse reaction to another vaccine;

YES NOT APPLICABLE NO

6. Which sources of information do you trust the most?

- Healthcare workers
- Internet
- Social network
- Media
- People close to you

7. Would you receive the HPV vaccine after pharmacy counselling service?

- Yes
- I am not sure
- Not now
- No
