# Supplementary material for: Strengthening the role of community pharmacy in HPV vaccination roll-out in Serbia at national and local levels: A pharmacy-based education approach
Source: PLoS One. 2025 Apr 29;20(4):e0322584. doi: 10.1371/journal.pone.0322584 (PMC12040191; doi:10.1371/journal.pone.0322584)
Supplement: S2 Material — (PDF) [file pone.0322584.s002.pdf]

Questionnaire for parents/guardians

1. Has the child already received the HPV vaccine?

Yes.   No

If "NO", provide counseling service. If the answer is "YES", record that answer and no further service is required.

2. Do you know what Human papilloma virus (HPV) is?

Yes Partially NO

Indicate what advice/information you have given to the service user:

a) What is HPV;

Yes   No

b) How HPV is transmitted;

Yes. No

c) How HPV infection can be prevent;

Yes. No

3. Do you know what problems HPV causes?

Yes Partially No

Indicate what advice/information you have given to the service user:

-a) What are the possible consequences of HPV infection;

Yes. No

b) Do all women/men with HPV infection get cancer;

Yes, No

c) How common is cancer caused by HPV infection;

Yes No

4. Do you know about the HPV vaccine?

Yes Partially No

Indicate what advice/information you have given to the service user:

a)   Why HPV vaccination is important;

YES. NO

b)   - Can both boys and girls be vaccinated;

YES. NO

c)   At what age a child can be vaccinated;

YES. NO

d) How many doses are needed in relation to age;
YES. NO

e) Whether the child should receive all the recommended doses of the vaccine;
YES. NO

f) What is the recommended time to schedule between two/three doses of the HPV vaccine;
YES. NO

g) How long protection lasts after complete HPV vaccination;
YES. NO

h) will the HPV vaccination prevent my child from getting cancer;
YES. NO

i) Whether my child can get an HPV infection after HPV vaccination;

YES. NO

j) Vaccination recommendation for young people who are not yet sexually active;
YES. NO

k) Where the HPV vaccine can be received;
YES. NO

l) Whether regular gynecological/urological examinations are required after vaccination;
YES. NO

m) the possibility of HPV vaccination of children with a disorder of the immune system/immunosuppressive disease;
YES NOT APPLICABLE NO

5. Are you concerned about the HPV vaccination of your child?

Yes Partially NO

Indicate what advice/information you have given to the service user:
The HPV vaccine is safe and effective;
YES. NO

b) - HPV vaccination does not affect fertility;
YES. NO

c) - the vaccine is equally effective in men and women;
YES. NO

d) contraindications for vaccination;
YES. NO

e) the reason for the possible occurrence of fainting after HPV vaccination;
YES. NO

f) the safety of vaccination if the child previously had an adverse reaction to another vaccine;
YES NOT APPLICABLE NO

6. Which sources of information do you trust the most?

- ○ Healthcare workers
- ○ Internet
- ○ Social network
- ○ Media
- ○ People close to you

7. Would you accept that your child (daughter/son), after providing counseling services at the pharmacy, receive the HPV vaccine:

- ○ Yes
- ○ I am not sure
- ○ Not now
- ○ Ne
