# Supplementary material for: Strengthening the role of community pharmacy in HPV vaccination roll-out in Serbia at national and local levels: A pharmacy-based education approach
Source: PLoS One. 2025 Apr 29;20(4):e0322584. doi: 10.1371/journal.pone.0322584 (PMC12040191; doi:10.1371/journal.pone.0322584)
Supplement: S1 Table — (PDF) [file pone.0322584.s003.pdf]

Supplementary Table 2. The relationship between prior knowledge about HPV and the decision about vaccination.

Would you receive the HPV vaccine after pharmacy counselling service?								
	Young adults				Parents/guardians			
	Yes	No	Not now	I am not sure	Yes	No	Not now	I am not sure
**Do you know what HPV is?**	*				*			
**Yes**	45%	25%	27%	24%	45%	30%	29%	23%
**Partially**	43%	30%	47%	46%	44%	32%	50%	45%
**No**	12%	45%	26%	29%	12%	38%	21%	32%
**Do you know what problems HPV causes?**	*							
**Yes**	35%	20%	20%	22%	34%	27%	19%	18%
**Partially**	50%	31%	31%	49%	53%	33%	56%	48%
**No**	14%	48%	48%	29%	13%	40%	24%	34%
**Do you know about the HPV vaccine?**	*				*			
**Yes**	51%	32%	37%	27%	47%	33%	30%	25%
**Partially**	28%	15%	30%	31%	35%	24%	36%	35%
**No**	21%	53%	33%	42%	19%	43%	33%	40%
**Are you concerned about the HPV vaccination?**	*				*			
**Yes**	14%	22%	15%	16%	15%	61%	29%	37%
**Partially**	33%	24%	46%	46%	40%	17%	56%	50%
**No**	53%	54%	40%	38%	45%	22%	15%	13%
**Which sources of information do you trust the most?**	*				*			
**Social network**	1%	7%	1%	4%	0%	4%	1%	2%
**Internet**	2%	11%	4%	7%	1%	10%	2%	5%
**Media**	0.5%	2%	1%	2%	0%	5%	1%	3%
**People close to you**	5%	26%	8%	8%	3%	34%	8%	11%
**Healthcare workers**	91%	53%	85%	79%	96%	46%	87%	79%

* Significant relationships between variables (p<0.01).
